# 2565. In vitro synergistic bacterial killing of imipenem and ceftaroline against *Mycobacterium abscessus*; a static concentration time-kill analysis

**DOI:** 10.1093/ofid/ofad500.2182

**Published:** 2023-11-27

**Authors:** Eunjeong Shin, Mary Nantongo, Steven M Holland, Barry N Kreiswirth, Khalid M Dousa, Robert A bonomo

**Affiliations:** Case Western Reserve University, Cleveland, Ohio; Case Western Reserve University, Cleveland, Ohio; National Institutes of Health, Bethesda, Maryland; Center for Discovery and Innovation, Hakensack Meridian Health, Nutley, New Jersey; Louis Stokes Cleveland Department of Veterans Affairs Medical Center, Cleveland, Cleveland, Ohio; Louis Stokes Cleveland Department of Veterans Affairs Medical Center, Cleveland, Cleveland, Ohio

## Abstract

**Background:**

*Mycobacterium abscessus* (*Mab*) represents a significant clinical challenge, with a rising incidence in recent times. The current therapeutic approach, which involves prolonged IV administration of amikacin, is associated with notable toxicities. Consequently, there is a pressing need to explore safe alternatives in antibiotic therapy. This study aimed to evaluate the *in-vitro* synergistic effect of a double β-lactam (DBL) combination which is safe for all ages.

**Methods:**

We conducted static concentration time-kill (SCTK) studies utilizing *Mab* strain ATCC19977 over 10 days in duplicate. SCTK evaluated the impact of imipenem (IPM) and ceftaroline (CFT) in both monotherapy and combination therapy on bacterial killing (inoculum: 6 log_10_ CFU/mL). To counteract the recognized extent of thermal degradation of β-lactams, predetermined quantities of IPM and CFT were added every 24 hours. Synergistic killing was estimated in the S-ADAPT software. In addition, the post-antibiotic effect (PAE) was measured following a 2-hour exposure to β-lactams. The β-lactams were removed by exchange with drug-free broth after centrifugation.

**Results:**

DBL of IPM+CFT exhibited synergistic bactericidal activity, surpassing that of monotherapy with rapid regrowth observed in 1-3 days. Monotherapy demonstrated up to ∼1.5 log_10_ killing followed by near-complete regrowth over a period of 10 days. DBL demonstrated significant synergistic killing, resulting in rapid and the most extensive reduction (∼3.5 Log_10_ CFU/mL) and suppression of regrowth over 10 days. The addition of a β-lactamase inhibitor reduced the CFT concentration required for bacterial killing, resulting in synergistic bacterial killing at an achievable concentrations *in-vivo* mouse studies and clinical trials in the future. Furthermore, PAE were 4 minutes for IPM and 2 hours for CFT. Strikingly, the DBL at a concentration of 1xMIC demonstrated noteworthy bacteriostatic activity, with the suppression of bacterial growth lasting up to 14 h.

**Figure:**
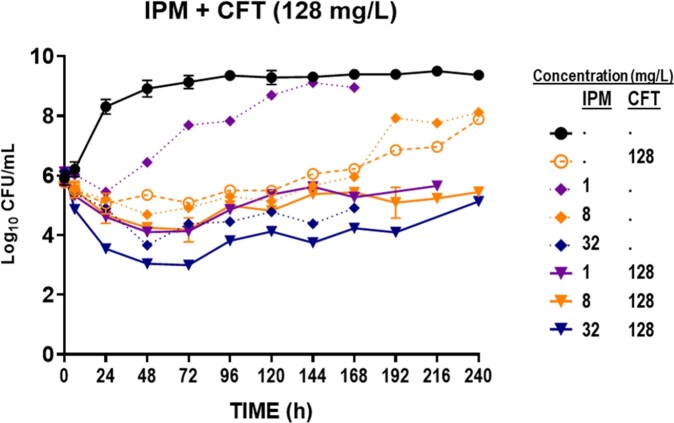

**Conclusion:**

DBL therapy yielded the most rapid and extensive killing of *Mab* with limited regrowth. The β-lactamase inhibitor was efficacious in inhibiting a broad-spectrum β-lactamase (*bla*_Mab_). The prolonged PAE observed in the DBL of IPM plus CFT may prove beneficial for intermittent dosing regimen.

**Disclosures:**

**Robert A. bonomo, MD**, Entasis, Merck, VenatoRx, Wockhardt: Grant/Research Support

